# Effect of static magnetic field on DNA synthesis: The interplay between DNA chirality and magnetic field left‐right asymmetry

**DOI:** 10.1096/fba.2019-00045

**Published:** 2020-03-07

**Authors:** Xingxing Yang, Zhiyuan Li, Tatyana Polyakova, Alexandr Dejneka, Vitalii Zablotskii, Xin Zhang

**Affiliations:** ^1^ High Magnetic Field Laboratory Key Laboratory of High Magnetic Field and Ion Beam Physical Biology Hefei Institutes of Physical Science Chinese Academy of Sciences Hefei China; ^2^ Science Island Branch of Graduate School University of Science and Technology of China Hefei China; ^3^ Institute of Physics of the Czech Academy of Sciences Prague Czech Republic; ^4^ Institutes of Physical Science and Information Technology Anhui University Hefei China

**Keywords:** biomagnetic effects, DNA synthesis, homochirality, left-right asymmetry, magnetic field

## Abstract

Interactions between magnetic fields (MFs) and living cells may stimulate a large variety of cellular responses to a MF, while the underlying intracellular mechanisms still remain a great puzzle. On a fundamental level, the MF — cell interaction is affected by the two broken symmetries: (a) left‐right (LR) asymmetry of the MF and (b) chirality of DNA molecules carrying electric charges and subjected to the Lorentz force when moving in a MF. Here we report on the chirality‐driven effect of static magnetic fields (SMFs) on DNA synthesis. This newly discovered effect reveals how the interplay between two fundamental features of symmetry in living and inanimate nature—DNA chirality and the inherent features of MFs to distinguish the left and right—manifests itself in different DNA synthesis rates in the upward and downward SMFs, consequently resulting in unequal cell proliferation for the two directions of the field. The interplay between DNA chirality and MF LR asymmetry will provide fundamental knowledge for many MF‐induced biological phenotypes.

## 1 INTRODUCTION

**Figure 1:**
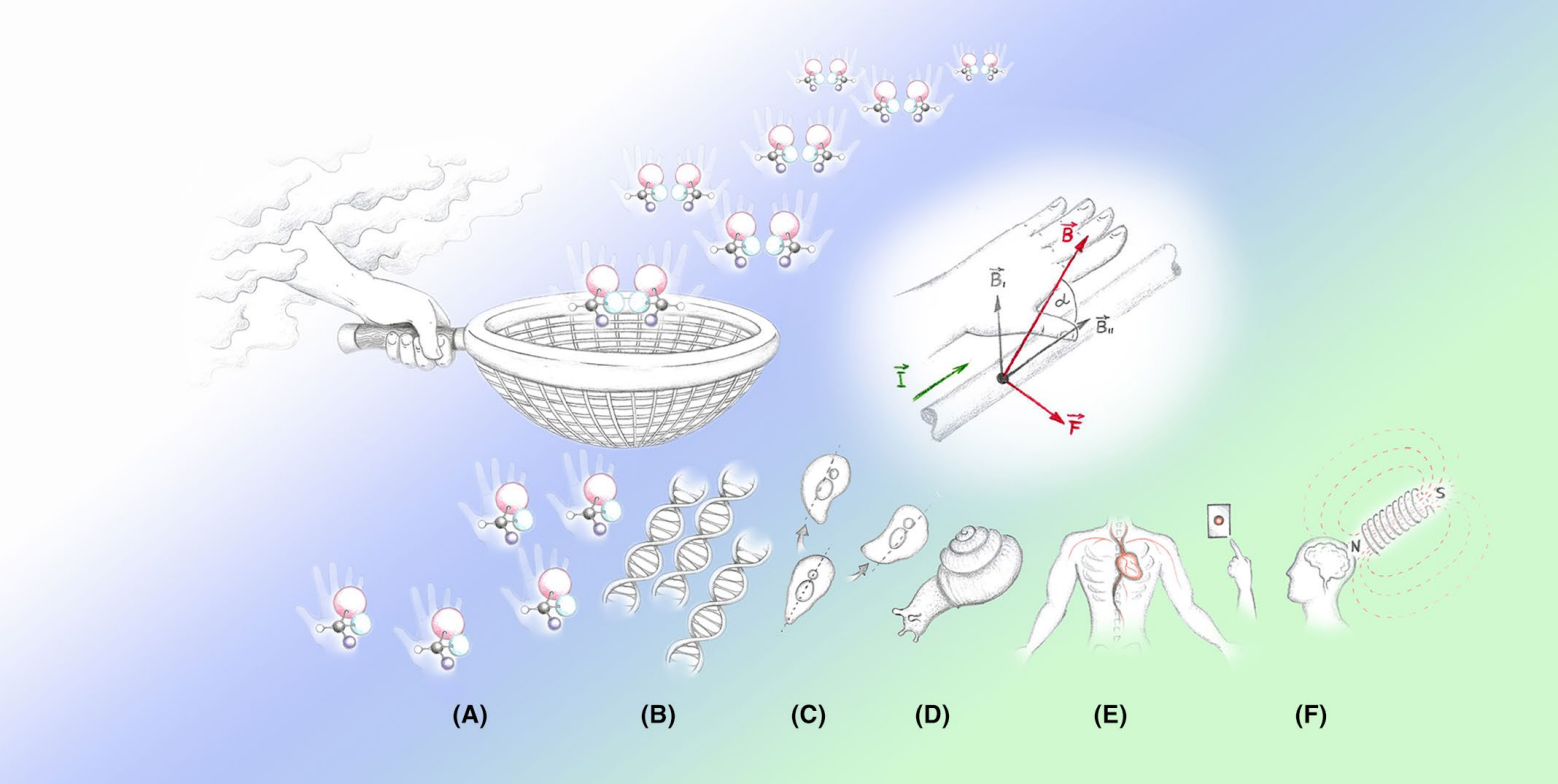
Chirality selection for life. On the bottom: (A) homochiral molecules of the left‐handed alanine, (B) DNA right‐handed helix, (C) LR asymmetry of cell division,^18^ (D) right (typical) form of the snail *Fruticicola lantzi* which is more viable than the inverse form,^24^ (E) LR asymmetry of human body and (F) LR inversion in the human brain under influence of a magnetic field (see the text below). Importantly, all amino acids are present in all proteins only in the left configuration. It has been proposed that the amino acids (in particular, left‐handed alanine) chiral selection takes place in strong MFs generated by neutron stars, for example, see Ref. 69

One of the long‐standing unsolved problems in biology has been the search for mechanisms of interactions between MFs and living cells and organisms. Despite the fact that many intriguing mechanisms have been suggested,^1^, ^2^, ^3^, ^4^, ^5^, ^6^, ^7^, ^8^ a fundamental aspect of the interaction between MF and cell—inherent DNA chirality and MF left‐right (LR) asymmetry—has not been yet explored. Living organisms consist of chiral molecules that can exist in two mirror‐symmetric forms: right‐handed or left‐handed enantiomers. At the same time, in the entire biosphere, substances that constitute the molecular life basis and carry the pivotal life functions are found in organisms in only one of these two forms. It is an important feature of life processes to establish the chiral purity by preventing or eliminating one of the enantiomer's form. The homochirality (LR asymmetry) is an inherent property of living matter and its source and assignment are poorly understood and represent the subject of many hypotheses.^9^, ^10^ For example, according to the Vester‐Ulbrict hypothesis, the longitudinal polarization of cosmic beta radiation was responsible for the origins of biological homochirality.^11^, ^12^ It is possible that both DNA chirality and asymmetrical behavior of DNA with supercoils of opposite signs exerted physical constraints and contributed to early choices for biological homochirality in the nascent life.^13^, ^14^ Thus, many hypotheses of the origin of life suggest that in order for life to emerge, something first had to crack the symmetry between left‐handed and right‐handed molecules,^15^ which is illustrated in Figure 1. In living organisms, the chirality manifests itself not only in primary building block molecules, but also in more complex formations: protein, cell, and embryonic development.^16^, ^17^

Chirality can also be observed as the directional rotation of cellular organelles, cytoskeleton, and cells as a whole.^18^ Findings^19^, ^20^, ^21^, ^22^, ^23^ suggest that chirality is a fundamental property of the cell that depends on the chiral nature of the mitotic spindle and cytoskeleton network, such as actin and microtubule bundles. It is believed that all amino acids are present in all proteins only in the left configuration. Nucleotides—the basic structural elements of RNA and DNA nucleic acids—contain only the right configuration of ribose sugar. Other major sugars included in the polysaccharides, such as glucose and fructose, are found only in the right configuration, and rhamnose sugar is only in left.^24^ Moreover, many of the chemical reactions that drive living cells only deal with molecules of the correct handedness. The effects of the LR asymmetry manifest themselves in a wide variety of vital functions of organisms and human right down to the sphere of the psyche. For example, visual perception of Raphael's *Sistine Madonna* changes significantly on reflection in a mirror.^24^, ^25^ Such a clearly expressed LR asymmetry in living systems, which is not observed in the inorganic world, until now seems somewhat mysterious or, in any case, difficult to explain.

**Figure 2:**
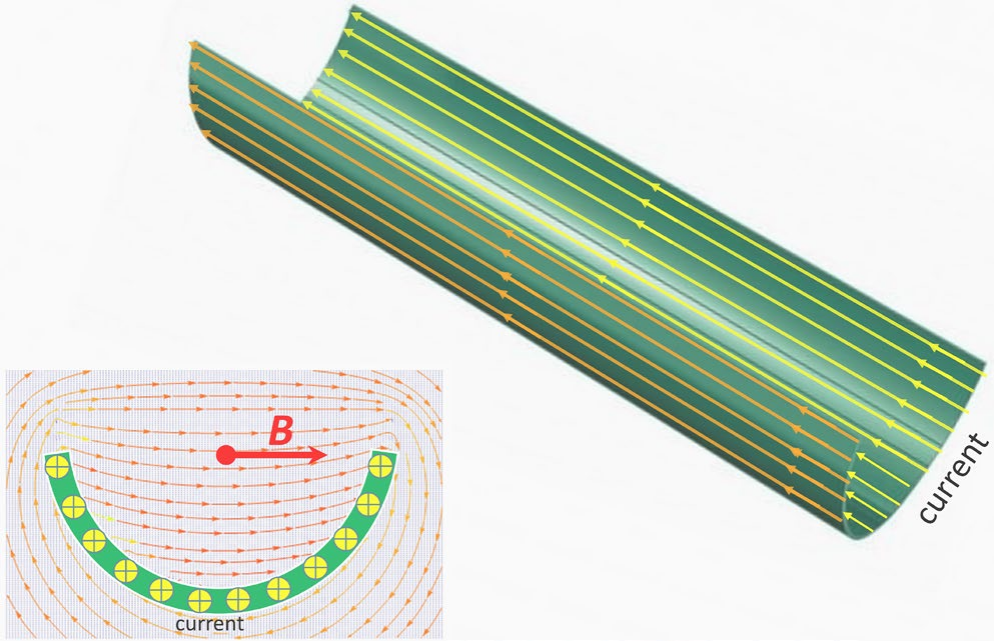
LR asymmetry—“breaking the mirror”—of MF generated by the current flowing along a thin wall infinite gutter. In the gutter’s wall, the current is uniformly distributed with the density per unit length, *i* = *I*/π*R* (where *R* is the gutter radius, and *I* is the current). The current is perpendicular to the plane of the left drawing, as shown by the circles with crosses. The red lines with arrows represent the calculated vector field of the magnetic induction (*B*). Despite the fact that the whole system is mirror‐symmetric about the vertical plane passing through the gutter center, the MF of the current prefers the right direction indicated by the red arrows. The value of the magnetic induction at the gutter axis is *B* = *μ*_0_ *i*/π. LR magnetic field symmetry breaking is evident in the weak interaction (Yang and Lee the 1957 Nobel Prize in Physics)

Even more surprising is the fact that the LR asymmetry in life processes can be somehow connected with LR asymmetry of—the direction of the MF of an infinitely long wire with uniform current is determined by the right‐hand grip rule (a more sophisticated case is shown in Figure 2). For example, the LR asymmetry could be created during embryonic development by an electric current running down the length of the notochord and generating a MF vector pointing either R or L.^26^ Although the relationship between the LR asymmetry of the MF and the dissymmetry of the main living substances are apparently ambiguous and far from unequivocal, there are some interesting experimental evidences indicating their potential links. For example, at human body level, MF stimulation to the left posterior parietal cortex induces an LR inversion in the human brain.^27^ People's brain alpha were studied to determine whether the brain reacts to changes in MF direction.^28^ This study poses an intriguing question—why people seem to respond to downward‐ but not upward‐pointing MFs. Upward and downward MF directions produce divergent effects on cancer cell numbers at cellular level as well as tumor growth in mice.^29^

We see that the problem of the chirality of molecules in living organisms and their interactions with MF represents a very versatile problem of physics, chemistry, and biology, which has not been resolved so far. In this work we attempt to shed a light on this unsolved mystery by studying effects of static magnetic fields (SMFs) on DNA synthesis in various types of cells. We first highlighted the importance of physical principles and symmetry laws in fundamental biological processes. A model of DNA rotation in an SMF is presented in Section 2. Section 3 shows the experimental results of the SMF effect on DNA synthesis in four cell lines. Mechanisms of dysregulation of DNA replication by SMF and topotecan are discussed in Section 4. The achieved results and prospects for further research are discussed in Section 5.

## 2 MODEL OF DNA ROTATION IN SMF

**Figure 3:**
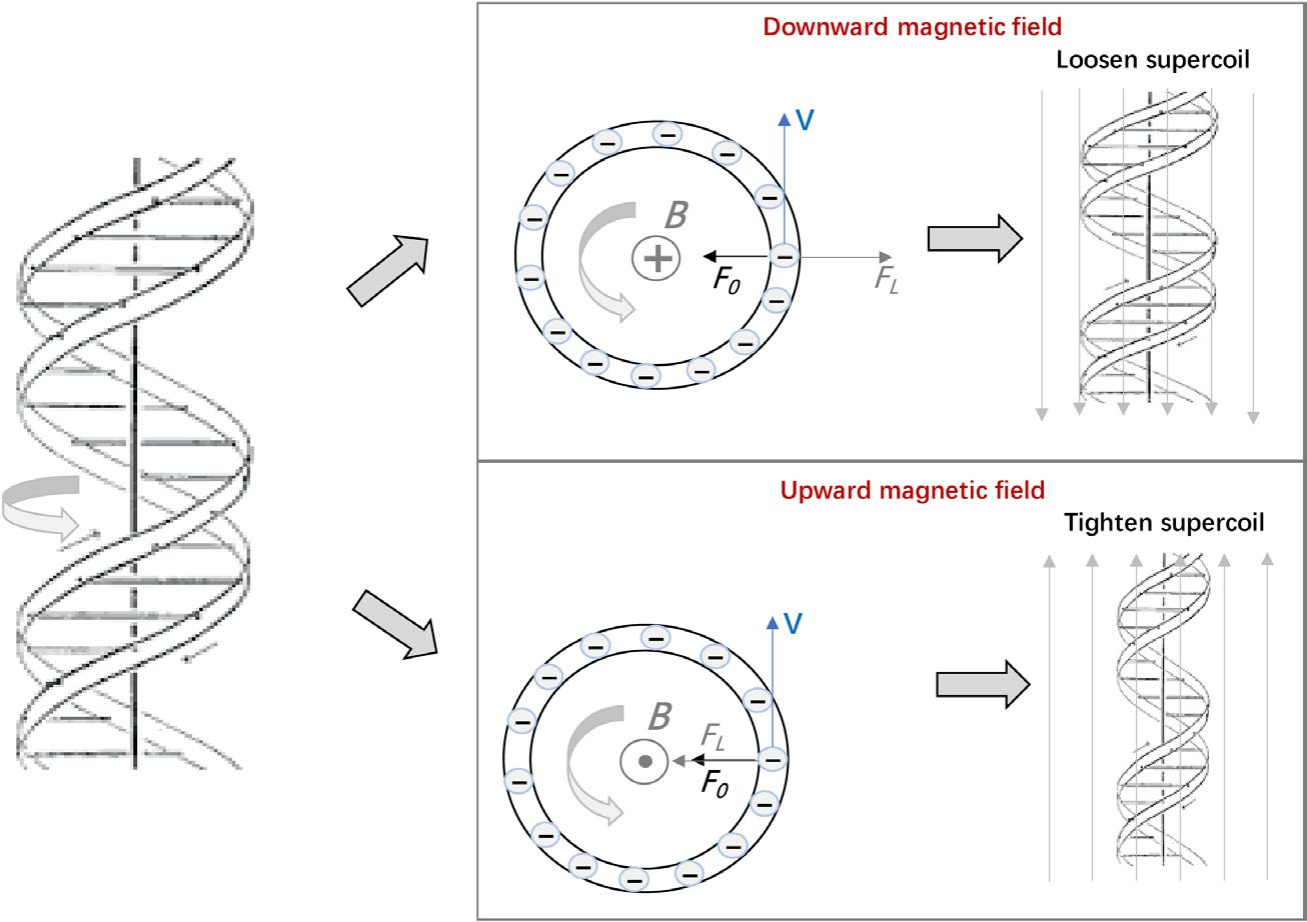
Cranked DNA motion and the magnetic Lorentz forces, (left) side view of DNA, (right) top view of DNA cross section. For downward and upward MF, the Lorentz force (*F*_L_) of negatively charged DNA has different directions

The nature of intertwined double strand DNA determines that the DNA has to rotate in cells.^30^ Let us consider DNA that rotates in an SMF with induction *B*. We assume that in the absence of MF, replicating DNA rotates with the angular velocity *ω*_0_. This is so‐called cranked DNA motion,^31^ or in the other words, plumber's snake motion^32^ (Figure 3, on the left). In the absence of MF, DNA rotation is described by.

(1) $$m\omega_{0}^{2}\;R=F_{0}$$

where *F_0_* is an endogenous centripetal force determining DNA rotation, *m = ρ πa^2^L* is the DNA‐fragment mass, *R* is its rotation radius, *ρ* is the DNA mass density, *L* is the DNA‐fragment length and *a* is the DNA radius. When MF is switched on parallel to the DNA helix axis, the magnetic Lorentz force (*F*_L_) acts on the moving DNA’s negative charges distributed with the surface density, *σ*. For downward (Figure 3 the right, top) or upward (Figure 3 the right, bottom) SMFs, the equation of motion is

(2) $$m\omega^{2}\;R=F_{0}\pm qvB$$

where *B* is the MF induction, *q* = *σ*2π*aL* is the DNA fragment charge, the signs “+” and “−” are taken for upward and downward magnetic field directions, accordingly (Figure 3, on the right), *v* = ω*R* is the charge velocity. We emphasize that counterions, which form an ion atmosphere surrounding a DNA, do not rotate together with the DNA and therefore they do not subject to the Lorentz force from an SMF, while ion atmosphere attenuates the electric field of the negative charges of phosphoryl groups. Quantitatively this can be explained as follows. In ion atmosphere, positive ions are attracted to nucleic acids due to their negative charge and a region of high counterion density is localized to approximately 10 Å around the nucleic acid.^33^, ^34^ The electrostatic interaction energy between a nucleic acid group and a positive charge (+e) of the ion atmosphere at the distance 10 A is estimated to be 0.7 *k*_B_ *T*, which is less than the thermal fluctuation energy, *k*_B_ *T* = 4.1 pN·nm, where *k*_B_ is the Boltzmann constant and *T* is temperature. This implies that in the local environment of replicating DNA, the thermal fluctuations,^35^ rotational drag (which force is dozen of pN and torque of the order *k*_B_ *T* per 10 kbp DNA at 2000 turns per second^31^) and centrifugal force easily disrupt bonds between the nucleic acid groups and ion atmosphere.

The magnetic field induced change of the angular velocity, ∆*ω*_mag_ = |*ω*‐*ω*_0_| is obtained from Equations (1) and (2) as

(3) $$\Delta \omega_{\text{mag}}\approx \pm \frac{B\sigma }{\rho a}$$

Thus, the SMF switching leads to either acceleration or damping of the natural DNA rotation. The difference of the angular velocities is proportional to the MF induction and the charge density of DNA. Of note, Equation (3) was obtained regardless of viscous hydrodynamic friction acting on DNA rotating in medium. Observations^36^ suggested that the chiral hydrodynamic interactions between the asymmetric biomolecules and fluids are important in natural biological systems. DNA, when it forms a double‐stranded structure, can show hydrodynamic preference to a right‐handed vortex than to a left‐handed vortex.^36^ In the other words, hydrodynamic frictions are different for the right‐ and left‐handed torsional DNA flows. Regarding these friction forces, the SMF‐induced difference ∆*ω*_mag_ between left‐handed and right‐handed DNA rotations will be larger than that predicted by Equation (3). If an SMF is applied at an angle instead of parallel to the DNA helix axis the MF causes the DNA to precess around the direction of the MF with the frequency given by Equation (3). This is similar to the Larmor precession of a magnetic moment in magnetic field.

## 3 EXPERIMENTAL RESULTS

**Figure 4:**
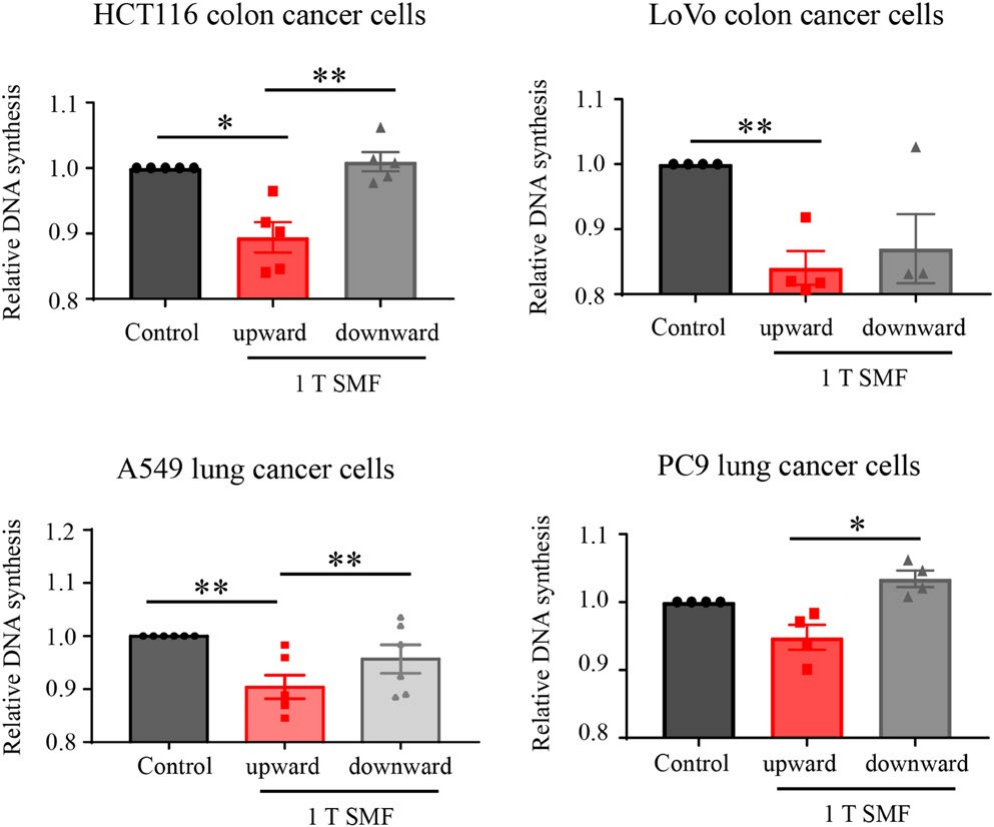
DNA synthesis is decreased by 1 T upward but not downward MF. Experiments were repeated for 4‐6 times for each cell line. 1 T SMF was generated by permanent magnets as previously described.^29^, ^65^, ^66^, ^67^ *P < .05, **P < .01. BrdU incorporation was used to measure DNA synthesis

In order to verify the theoretical calculation model, we chose two colon cancer and two lung cancer cell lines to detect the SMF effect on DNA replication in cells. DNA replication in cells was determined by BrdU incorporation, a widely used method to examine DNA replication using a nucleoside analog BrdU, in the presence or absence of moderate SMF. Since DNA replication occurs during S‐phase of the cell cycle, we used double thymidine block,^37^, ^38^ a widely used method to synchronize cell into G1/S border by DNA synthesis inhibition. The cells sequentially enter S‐phase after thymidine washout. Double thymidine block, which blocks the cell cycle with thymidine for two rounds, is commonly used to improve synchronization efficiency. For the second thymidine release, we used medium containing 10 µmol/L BrdU, in the presence or absence of 1 T SMF for 8 hours to evaluate the effect of SMF on DNA synthesis in S‐phase. Then the cells were harvested and fixed in 70% ice‐cold ethanol overnight at −20°C. The BrdU incorporation was determined by BrdU antibody and corresponding Alexa‐488 conjugated secondary antibody by flow cytometry. The DNA replication was evaluated by the mean fluorescence intensity of BrdU. We observed that the DNA replication was decreased by about 5%‐15% by upward direction SMF in four different cell lines, while downward direction SMF did not generate such effect (Figure 4). It should be noted that different types of cells exhibit different responses to SMF because this response depends on many factors, such as cell type, age, differentiation state, cell rigidity, cell polarity, and other external factors influencing the cell machinery.^39^

**Figure 5:**
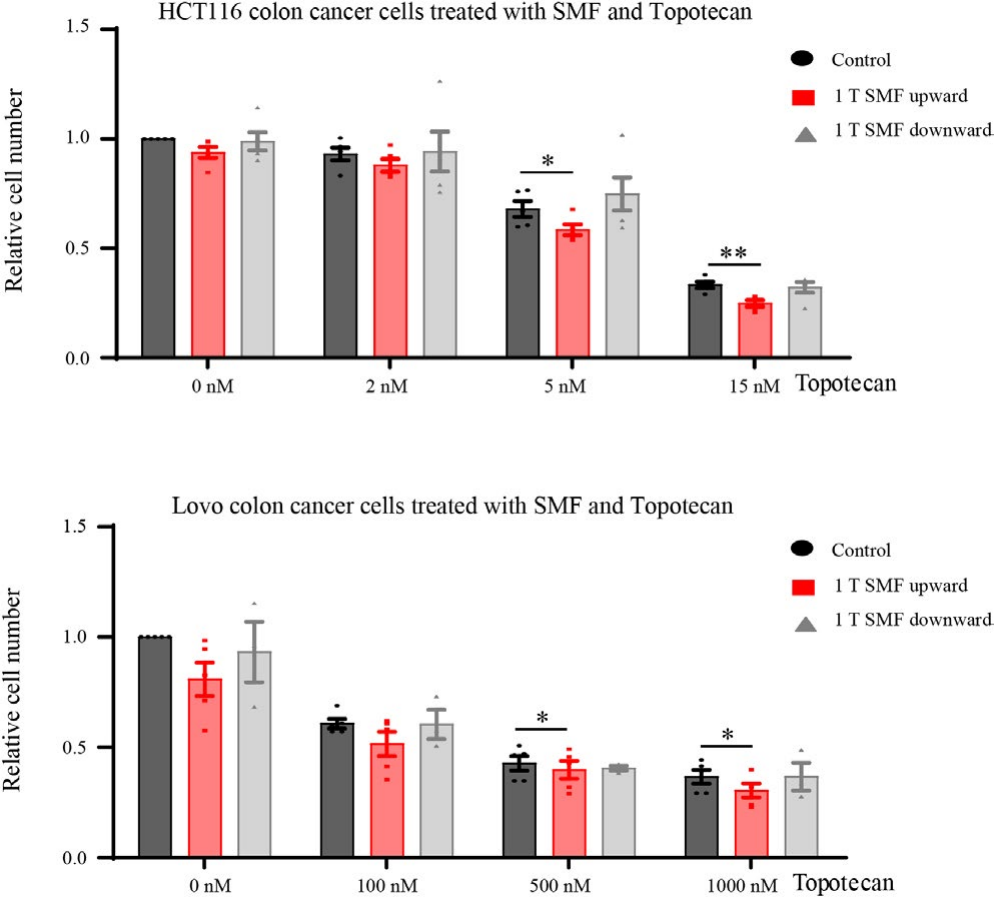
Topotecan has a combinational effect with the upward but not downward SMF for cell proliferation. HCT116 and LoVo cells were treated with 1 T SMF in combination with different concentrations of topotecan for 2 d before their cell numbers were counted. Experiments were repeated for at least three times. *P < .05, **P < .01

Since Topoisomerases could alter the supercoiling of double‐stranded DNA, we chose topotecan, a topoisomerase I inhibitor^40^, ^41^ that can interfere with supercoil relaxation, in combination with SMFs. Two colon cancer cell lines HCT116 and LoVo were plated first day and treated with corresponding concentrations of topotecan or DMSO as control for another 2 days before they were harvested for analysis. Since decreased DNA replication caused decreased S‐phase progression and cell proliferation, which led to reduced cell number, we used cell number counting for this experiment. Similar to most other drugs, different cell types have different sensitivity to topotecan. We found that 5 and 500 nmol/L of topotecan alone reduced HCT116 and LoVo cell number to around 70% and 40%, but when the cells were exposed to 1 T upward SMF, the cell numbers were further reduced for another 10% and 5%, respectively (Figure 5). These results indicate that supercoil relaxation effects of topotecan and 1 T SMF may have a combinational effect on DNA synthesis and cancer cell proliferation.

## 4 MECHANISM OF DYSREGULATION OF DNA REPLICATION BY SMF AND TOPOTECAN

Let us estimate using Eq.3 the difference between the angular velocities of the left and right DNA rotation in the presence of SMF for DNA of the radius *a* = 1 nm and *a* = 1.2 nm (for B‐ and A‐forms of DNA, accordingly), the mass density *ρ* = 1407 kg/m^3^,^42^ *B* = 1 T and the surface charge density *σ* = −0.15 C/m^2^.^43^, ^44^ By inserting these parameters into Equation (3), one can obtain ∆*ω*_mag_ ≈ ±(8.9‐10.7)×10^4^/s, which corresponds to (1.4‐1.7)×10^4^ turns per second. It is interesting to compare the estimated value of ∆*ω*_mag_ with the DNA natural angular velocities. For example, the rate of unwinding of the parent DNA in the replicative fork in *Escherichia coli* cells is 60 kb/min,^45^ which corresponds to angular velocity *ω*_0_ = 628/s. The balance of the driving torque (a typical driving torque of ~20 pN×nm) and rotational drag torque (estimated for viscosity of water) requires that DNA can maximally rotate about its axis at a frequency equal to 5×10^6^ turns per second for *L* = 50 nm, or at 2.5×10^5^ turns per second for *L* = 1 μm.^46^, ^47^ However, optical tweezer measurements of the rotational drag of a single DNA molecule gives drag torque of the order of 3.76 pN·nm for 10 000 base pairs of DNA and its rotation speed about 2000 turns per second,^31^ which corresponds to the angular velocity *ω*_0_ = 1.26×10^4^/s.

Comparing the estimated value ∆*ω*_mag_ with the values of *ω*_0_, one should keep in mind the two following facts. First, the rotation of the DNA inside the enzyme clamp is not free but is hindered by friction as shown by measurements.^48^ Therefore friction slows down the SMF‐induced DNA rotation rate compared to the unhindered rate. Second, a more sophisticated model of cranked DNA motion, for example, hybrid motion of a naturally bent semi‐flexible rod,^32^ would lead to a smaller effect of SMF on DNA rotation. Nevertheless, the above described model of the cranked rigid DNA rotation allows us to conclude that an SMF can alter the DNA replication speed. Indeed, since the estimated above ∆*ω*_mag_ has the same order or even larger than the DNA native angular velocity, SMF‐induced acceleration of DNA rotation can lead to breaking the speed limit of DNA replication and/or transcription, while SMF‐induced slowdown can pause DNA rotation and replication process. Thus, two limiting cases of SMF effect on DNA rotation can be drawn: breaking the speed limit of DNA replication and stopping DNA replication.

In intermediate cases, depending on the mutual orientation, an applied SMF, and DNA helix axis, the SMF can either accelerate or slow down DNA rotation during its replication. Slowing down DNA rotation causes a time delay of DNA replication. In contrary, an acceleration of DNA rotation does not necessarily lead to faster DNA replication and transcription because there are many other speed limiting mechanisms are also involved in this process.^49^ Moreover, acceleration of DNA rotation can lead to the generation of an additional number of negative or positive supercoils, which could also decrease the replication rate.^48^ Theoretically, an acceleration of DNA replication may also lead to additional errors in the following transcription. So, in both cases, MF added to DNA rotation (Equation [3]) could lead to cell death. In another word, an SMF destabilizes the replication machinery of DNA and could result in cell death.

*The role of a DNA topoisomerase is to resolve topological problems of DNA*. In particular, DNA topoisomerase prevents the supercoils and thereby provides an elongation of replicating DNA chains.^50^ Results^51^ indicate that human topoisomerase IIα relaxes positively supercoiled plasmids >10‐fold faster than negatively supercoiled molecules.

*The role of topotecan is to impede DNA uncoiling by topoisomerase I inhibition*. The dynamics of the DNA swivel in the presence of topotecan was analyzed.^52^ For example, DNA extension velocities: uncoiling with topotecan *V* = 0.2 mkm/s, while uncoiling without topotecan *V* = 3‐5 mkm/s.^52^ Of note, in the presence of topotecan the angular velocity difference between positive and negative supercoils is ∆*ω* = −10 to 0 Hz. Interestingly, that the LR asymmetry manifests itself here: positive supercoils were removed more slowly than negative supercoils.^46^

*Mechanisms of SMF action and its asymmetry*. Supercoils generation by DNA rotation in the presence of SMF is an essential point of the mechanism to be considered. We propose that SMF may affect the DNA replication as follows: (a) For the upward MF, the MF accelerates DNA rotation to tighten the supercoils or generate new supercoils, which could directly slow down DNA replication. Then if the topoisomerase can work properly, the supercoils could be relieved. However, in the presence of topotecan, the extra supercoils caused by upward direction SMF could impede DNA replication. (b) For the downward MF, it could potentially decrease the angular velocity of DNA rotation and loosen the DNA supercoils. This could potentially increase DNA replication because it opens up DNA structure, but it could also slow down DNA replication because DNA rotation/spin is a necessary step during DNA replication. These factors may add up and show differential phenotypes in different cell types.

In general, due to the random orientation of replicating DNAs, this effect of SMF on cell number has a statistical nature. Performing 3D orientation averaging, one can conclude that only one of six parts of cells is affected by a vertical SMF. The effect of SMF on the DNA replication rate is reinforced by topotecan. Indeed, the topotecan impedes DNA uncoiling by topoisomerase inhibition thereby shortening replicating DNA chains.^46^ The observed asymmetry in the cell number for the upward and downward SMFs is a consequence of (a) inherent asymmetry of DNA and its replication, (b) inherent LR asymmetry of MF, and (c) a preference of the vertical DNA orientation in nuclei, for example, due to gravity or MF (for a putative mechanism, please see the Discussion section).

At the onset of DNA replication, a DNA part begins to rotate, and the magnetic Lorentz force starts to act on the negatively charged DNA. If endogenous or other reasons for the appearance of supercoils arise, the magnetic force can select either the clockwise or counter‐clockwise directions. Thus, on DNA structure, either the positive or negative supercoils appear depending on the orientation of MF. Both DNA chirality and asymmetrical behavior of supercoiled DNA of opposite signs exert some physical constraints to DNA topology and unwinding.^53^ This implies that the total number of positive and negative supercoils will be different on DNAs subjected to up and down MFs. Of note, the positive and negative supercoils have the different relaxation times during DNA transcription.^46^, ^52^

Bearing in mind that different number of positive and negative supercoils together with their different relaxation times all affect DNA transcription rate, one can conclude that an application of either up or down SMF will finally lead to different expression and function of cell growth regulators, which potentially regulate the numbers of cells in the up‐ and down‐groups.

## 5 DISCUSSION

We have addressed the interplay between two fundamental features of symmetry in living and inanimate nature: (a) the broken LR symmetry of DNA and (b) the inherent feature of MFs to differ the left and right (see Figure 1 and the caption). Our proof‐of‐concept experiments demonstrate the moderate effects of MF‐induced dysregulation of DNA replication, when DNA chirality and MF right handedness interact in living cells. The effect manifests itself in the different rates of DNA synthesis in the upward and downward SMFs, resulting in different numbers of cells surviving in up‐ and down‐magnetic fields. In spite of the small effect, this may have a fundamental significance for our understanding of biological effects of MFs. The effect of magnetically induced dysregulation of DNA replication is observable in cancerous cells because of their accelerated proliferation rates.

The small observed effects are caused by the fact that applied SMF somehow interacts with the randomly distributed cell bodies and/or cell receptors and DNAs without any unidirectional anisotropy. However, it is natural to expect a small preference in the DNA orientation along the gravitational force. Most probably, the preference in vertical DNA orientation may arise during DNA replication. Indeed, gravity drives biological systems toward specific organization patterns.^54^ The gravitation (mechanical) forces being perceived by cellular receptors and then are directly or indirectly transmitted by a cellular mechanotransduction machinery to the cell nucleus and therein DNAs.^55^ While this is an intriguing mechanism that could potentially explain a combinational effect of topotecan and upward MF on the surviving cell, a number of important questions related to this mechanism remain to be addressed.

First of all, it is not clear whether a DNA tends to adapt a vertical position during replication and transcription, which presumably is a prerequisite for its inhibited replication. When cell enters mitosis (the so‐called "soft mode"^39^), it would be "physically reasonable" to expect a reorientation of cell organelles by a MF. For example, the orientation of early cleavages of Xenopus embryos and changes in cleavage‐furrow geometries were observed in strong (1.7‐16.7 T) SMFs. To explain the observed orientation of the mitotic apparatus, it was hypothesized that the MF acts directly on the microtubules of the mitotic apparatus.^56^ Another mechanism is that DNA orientation is related to the role of gravity. The role of gravity in the DNA orientation is determined by the relatively large mass density of DNA (appr. 1400 kg/m^3^), which somehow floats in chromatin solution inside the cell nucleus. Thus, from a mechanical point of view, DNA patterns are undoubtedly gravity sensitive. For example, gene expression in human T cells rapidly (20 seconds‐5 minutes) responds to altered gravity,^57^ which implies that cells are equipped with a robust and efficient adaptation potential when challenged with altered gravitational environments. In plants, molecular mechanisms of the gravitropism are related to amyloplasts, which has the mass density of 1500 kg/m^3^ whereas the surrounding cytoplasm has the density of approximately 1020‐1100 kg/m^3^.^58^ Thus, one can suppose that in cell nuclei a statistical preference in vertical alignment of DNA is mainly driven by gravity. It is interesting that in plants a moderate SMF and gravity, working together, are capable of synergistically coordinating the direction of the root growth. It was recently shown that 600 mT SMF regulates the root growth by altering CRY and auxin signaling pathways in Arabidopsis.^59^

Secondly, we hypothesize that SMF could align the DNA/chromatin parallel to its direction and exert Lorentz forces on rotating DNAs to affect their tightness, but we do not have direct experimental evidences for this due to technical limitations. However, our current paper focuses on SMF effect on DNA synthesis and there are multiple evidences supporting this hypothesis. For example, it is already known that DNA chain has relatively large diamagnetic anisotropy^60^ and theoretical predictions suggested that mitotic chromosome arms might generate electromagnetic fields along the chromosome arm direction^61^ and chromosomes theoretically should be fully aligned by SMFs of around 1.4 T.^62^ We have previously shown that ultra‐high SMF up to 27 T could affect the orientation of spindles in the cell after 4‐hour exposure, which is determined by both microtubules and chromosomes.^63^ Although different methods could be used for the alignment of individual DNA molecules, all these methods consider the immobilization and/or constriction of DNA by either chemically modified surfaces or geometry (pores, channels etc). As for the possibilities to use DNA fibers to test our hypothesis, it was recently demonstrated that a single chromatin fiber is torsionally softer than a braided one by direct torque measurements.^64^ Since SMF directly interferes with torsional mechanics of DNA, experiments with DNA fibers will not help to test our hypothesis. Moreover, in a single oriented molecule experiment, there are no reasons for DNA rotation and replication under intrinsic forces because it is not a living system. In another word, a single oriented DNA molecule is not likely to rotate and replicate, which are the main subject of study in the present work. To test our hypothesis, DNA should be in a living cell, where during replication, DNA rotates under endogenous forces that are assisted or opposed by the Lorentz forces from SMFs. Lastly, it should be mentioned that while our work was under review process, a paper was published showing effects of SMF on plant depend not only on the MF intensity but also on its direction.^59^ We hope that our work could set the stage for probing SMF effects on DNA replication and synthesis.

In summary, our finding shows that a moderate SMF can dysregulate DNA replication and this effect is more pronounced in a specific case of the vertical direction of SMF. In our view, the principal interest lies in the demonstration of a synergistic effect of the LR asymmetry of MF and DNA chirality on DNA synthesis resulting in MF‐induced cell proliferation inhibition. To a great extent, the revealed magnetic targeting DNA‐dysregulation transcription pathway opens the door to develop new anti‐cancer therapy. Besides this, understanding the effects of MFs on life will provide the fundamental background necessary to understand the evolution of life forms.

## 6 METHODS

### 6.1 Cell culture

HCT 116, LoVo and A549 cells were cultured in DMEM without L‐glutamine (15‐017‐CVR), supplemented with 1% (v/v) GlutaMAX (35050‐061, Gibco), 10% (v/v) FBS (fetal bovine serum, FB25015, CLARK Bioscience), and 1% (v/v) P/S (penicillin/streptomycin, SV30010, HyClone), and PC9 cells were cultured in RPMI‐1640 without L‐glutamine (15‐040‐CVR, Corning) supplemented with 10% FBS, 1% GlutaMAX, and 1% P/S. All cells were cultured in 5% CO_2_, 37°C incubator.

### 6.2 Reagents

5‐bromo‐2′‐deoxyuridine (BrdU, 000103) and Alexa Fluor 488 (#A‐21202) were purchased from Thermo Fisher Scientific. Thymidine (T9250) was from Sigma. Na_2_B_4_O_7_ (Na_2_B_4_O_7_·10H_2_O,1303‐96‐4) was from Sangon Biotech. Anti‐BrdU antibody (#5292S) was purchased from Cell Signaling Technology. PI/RNase staining buffer (550825) was from BD pharmingen.

### 6.3 DNA synthesis assay

4 × 10^5^/mL cells plated in a 3.5‐cm dish on the first day were synchronized by double thymidine block. Briefly, cells were firstly blocked with 2.5 mmol/L thymidine in DMEM complete medium for 16 hours and then released for 8 hours in fresh DMEM complete medium containing 10 μmol/L 5‐bromo‐2′‐deoxyuridine (BrdU, 000103, Thermo Fisher Scientific) before washing with prewarmed phosphate‐buffered saline (PBS) three times. Then a second thymidine block for another 16 hours was performed to arrest cells into G1/S border. Sixteen hours later the cells were washed three times by prewarmed PBS and then maintained in DMEM complete medium with 10 μmol/L BrdU for another 8 hours exposed on 1 T SMF or not for control. Finally, the cells were trypsinized and washed by PBS before they were fixed in 70% ice‐cold ethanol overnight at −20°C. The fixed cells were washed by PBS and resuspended by 2 mol/L HCl, incubated at room temperature (RT) for 30 minutes on a rotator at 15 rpm/min. Centrifuged at 1833 *g* for 5 minutes, and resuspend cell precipitate in 0.1 mol/L Na_2_B_4_O_7_ (PH 8.5) at RT 10 minutes, centrifuged again, and washed the cells by PBS. Incubated the cells with the anti‐BrdU antibody (mouse, 1:200, #5292S, Cell Signaling Technology) at RT 2 hours in 50 µL staining buffer (TBS‐Tx supplemented with 2% BSA and 0.05% sodium azide), then cells were centrifuged and washed twice by TBS‐Tx (TBS supplemented with 0.1% Triton X‐100). The secondary Alexa Fluor 488‐conjugated antibodies (1:250, #A‐21202, Thermo Fisher Scientific) were incubated at RT for 1.5 hours and washed by TBS‐Tx twice. Finally, the cells were stained with PI/RNase staining buffer (BD pharmingen) for 15 minutes at room temperature in the dark and analyzed with flow cytometry (CytoFLEX, Beckman Coulter).

### 6.4 Magnetic field exposure

1 T SMF was provided by permanent magnets and the detailed magnetic configuration has been described before.^29^, ^65^, ^66^, ^67^, ^68^ Briefly, the magnets and the cells are all in a full‐sized CO_2_ cell incubator (Shanghai Boxun, BC‐J160S) that has accurate control of temperature (37°C), humidity and CO_2_ (5%). Cells were placed directly on the top surface of 5 × 5 × 5 cm neodymium N38 permanent magnets, with a measured magnetic field intensity of 1.07 ± 0.037 T by the Gauss meter (LakeShore 475 DSP Gaussmeter). The control group was placed at least 30‐40 cm away from the magnet with a measured magnetic field intensity of 0.925 ± 0.206 Gs in the same cell incubator to minimize the experimental variations. The experiments were repeated at least three times.

### 6.5 Topotecan treatment

Topotecan Hydrochloride (HY‐13768A) was purchased from MedChem Express, and stock solution was made by dissolving topotecan in DMSO at 20 mmol/L. 4 × 10^5^/mL cells were plated on the 96 plate first day, and 16 hours later the cells were treated with topotecan at a specific concentration or DMSO as control,^40^, ^41^ then exposed to 1 T SMF or not for another 2 days before they were harvested for cell counting. At the end of experiment, the cells were trypsinized by 100 μL trypsin and terminated by adding 100 μL medium, then analyzed with flow cytometry (CytoFLEX, Beckman Coulter).

### 6.6 Statistical analysis

In this manuscript, all experiments were repeated independently at least three times, and the data were analyzed by GraphPad Prism 8. Mean values are shown in the manuscript, and SEMs are shown as error bars. Student's *t* test was used to analyze the data of two groups, *P* values < .05 were considered as statistically significant.

## CONFLICT OF INTEREST

The authors declare no conflicts of interest.

## AUTHOR CONTRIBUTIONS

X. Zhang and V. Zablotskii suggested the original idea, designed research and drew the model; X. Yang and Z. Li performed cellular experiments; T. Polyakova, A. Dejneka, and V. Zablotskii performed the theoretical calculations. All authors analyzed data and wrote the paper.
